# Utility of the mFI-5 as a predictor of post-operative outcomes following gastrectomy for gastric cancer: an ACS-NSQIP analysis

**DOI:** 10.1007/s00464-024-11103-3

**Published:** 2024-07-24

**Authors:** Ashley Tran, Luke R. Putnam, John C. Lipham, Sharon Shiraga

**Affiliations:** Division of Upper GI and General Surgery, Department of Surgery, Keck School of Medicine of University of Southern California, 1510 San Pablo St., Suite 514, Los Angeles, CA 90033 USA

**Keywords:** Gastrectomy, Gastric cancer, Frailty, Frailty index, mFI-5, Complications

## Abstract

**Background:**

Gastric cancer is the 5th most common malignancy worldwide. Surgical treatment for the disease can often be highly morbid, especially in elderly patients. The modified 5-item frailty index (mFI-5), a recently developed tool for assessing patient frailty, has been shown to be an effective predictor of post-operative outcomes in various surgical fields. This study aims to assess the utility of the mFI-5 in predicting adverse postoperative outcomes following gastrectomy for gastric cancer.

**Methods:**

The National Surgical Quality Improvement Program (NSQIP) database was queried for patients who underwent partial or total gastrectomy for gastric cancer between 2011 and 2021. The mFI-5 score was calculated based on the presence of hypertension, congestive heart failure, diabetes mellitus, chronic obstructive pulmonary disease, and partially or fully dependent functional status. Patients were stratified into 3 groups according to mFI-5 score (mFI-5 = 0, mFI-5 = 1, mFI-5 ≥ 2). Univariate analysis and multivariate logistic regression were used to evaluate the association between mFI-5 score and post-operative outcomes.

**Results:**

7438 patients were identified (mFI-5 = 0: 3032, mFI-5 = 1: 2805, mFI-5 ≥ 2: 1601). mFI-5 ≥ 2 was an independent predictor of overall complications (OR 1.43, *p* < 0.001), serious complications (OR 1.42, *p* < 0.001), pneumonia (OR 1.43, *p* = 0.010), MI (OR 2.91, *p* = 0.005), and readmission within 30 days (OR 1.33, *p* = 0.008). Patients with higher frailty were more likely to experience unplanned intubation (OR 2.06, *p* < 0.001; OR 2.47, *p* < 0.001), failure to wean from the ventilator (OR 1.68, *p* = 0.003; OR 2.00, *p* < 0.001), acute renal failure (OR 3.25, *p* = 0.003; OR 3.27, *p* = 0.005), 30-day mortality (OR 1.73, *p* = 0.009; OR 1.94, *p* = 0.004), and non-home discharge (OR 1.34, *p* = 0.001; OR 1.74, *p* < 0.001) relative to non-frail patients.

**Conclusion:**

Higher frailty, as indicated by an increased mFI-5 score, raises the risk of serious post-operative complications in patients with gastric cancer undergoing gastrectomy. The mFI-5 has the potential to help identify high-risk patients and enhance pre-operative discussions and optimization.

**Graphical abstract:**

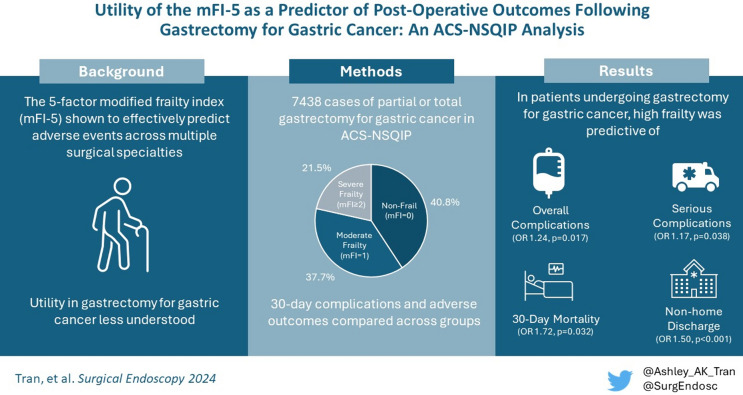

Despite an overall decrease in incidence in the United States and Western Europe, gastric cancer remains a major health problem globally [1, 2]. It is the fifth most common malignancy and third leading cause of cancer-related deaths worldwide [1–3]. Patients with early gastric cancer are often symptom-free [2, 4, 5]. Therefore, a majority of patients present with advanced disease [4, 5]. However, for patients who present with localized or locoregional, resectable gastric cancer, the first line curative treatment is endoscopic or surgical resection [4].

Endoscopic resection can be considered for very early gastric cancers (Tis or T1a) if they are confined to the mucosa, well-differentiated, ≤ 2 cm, and non-ulcerated [2, 4]. Patients that do not meet these criteria should undergo surgery. The extent of surgical resection is determined by multiple factors, including tumor location, TNM stage, and histopathology [2, 6]. Adequate gastric resection, which may entail proximal and distal gastrectomy, subtotal gastrectomy, or total gastrectomy, should achieve negative microscopic margins along with lymphadenectomy [6].

Despite being the primary curative treatment option for gastric cancer, patients undergoing gastrectomy are at risk for several serious complications including surgical site infections, anastomotic leaks, duodenal/pancreatic/lymphatic fistulas, post-gastrectomy syndromes, and esophageal strictures [7, 8]. Therefore, identifying patients who are at increased risk for post-operative complications and who may require prehabilitation prior to surgery is important for improving post-operative outcomes.

Frailty, a condition characterized by increased vulnerability to stressors resulting from a decline in functioning across multiple physiological systems, has been shown to be strongly associated with adverse outcomes, including falls, hospitalizations, and mortality [9, 10]. Several metrics have been proposed to objectively measure frailty. One of the first tools to be adopted was the Canada Study of Health and Aging Frailty Index (CSHA-FI), a 70-item scale based on factors such as cognitive function, nutritional status, and comorbidities [11].

Subsequently, the 11-facter modified frailty index (mFI-11) was developed, which contained 16 variables included in the American College of Surgeons National Surgical Quality Improvement Program mapping to the original CSHA-FI [11]. However, over time, the NSQIP variables have changed and many of the variables included in the mFI-11 are no longer reported. As such, the mFI-5 was developed [11]. This tool utilizes only 5 variables—history of congestive heart failure (CHF) within 30 days of surgery, diabetes mellitus, history of chronic obstructive pulmonary disease (COPD), non-independent functional status at the time of surgery, and hypertension requiring medication—and has since been shown to effectively predict adverse outcomes across multiple surgical subspecialties [11–16]. The mFI-5 has been used in smaller, single-institution retrospective studies to predict post-gastrectomy outcomes for gastric cancer patients [17, 18]. To our knowledge, there have been no large, multi-institution studies analyzing the utility of the mFI-5 in predicting adverse outcomes in this patient population. Therefore, the aim of this study was to evaluate the predictive ability of the mFI-5 by analyzing the association between mFI-5 score and 30-day adverse outcomes following gastrectomy for gastric cancer.

## Methods

### Patient selection

The 2011–2021 National Surgical Quality Improvement Program (NSQIP) was queried for patients undergoing partial or total gastrectomy for gastric cancer using appropriate Current Procedural Terminology (CPT 43611, 43,620, 43,621, 43,622, 43,631, 43,632, 43,633, and 43,634) and International Classification of Diseases (ICD-9 or ICD-10) codes (ICD-9 151.0, 151.1, 151.2, 151.3, 151.4, 151.5, 151.6, 151.8, 151.9 and ICD-10 C16.0, C16.1, C16.2, C16.3, C16.4, C16.5, C16.6, C16.8, C16.9). Patients with no available data on mFI-5 score variables were excluded.

### mFI-5 score calculation

The mFI-5 score was calculated for each patient based on the presence of the following comorbidities: history of congestive heart failure (CHF) within 30 days of surgery, diabetes mellitus, history of chronic obstructive pulmonary disease (COPD), non-independent functional status at the time of surgery, and hypertension requiring medication. The patient received 1 point for each comorbidity present, with a maximum score of 5 and minimum score of 0. Patients were stratified into three groups according to mFI-5 score: non-frail (mFI-5 = 0), mild frailty (mFI-5 = 1), and moderate-to-severe frailty (mFI-5 ≥ 2).

### Outcomes and definitions

Rates of 30-day postoperative complications, overall complications, serious complications, 30-day mortality, reoperation, readmission, and non-home discharge were analyzed. Overall complications was defined as the presence of any post-operative complication within 30 days of surgery. Serious complications was defined as organ space surgical site infection (SSI), deep incisional SSI, wound disruption, pneumonia, unplanned intubation, pulmonary embolism, failure to wean from the ventilator for over 48 h, acute renal failure (ARF), cerebrovascular accident/stroke with neurological deficit (CVA), cardiac arrest requiring cardiopulmonary resuscitation (CPR), myocardial infarction (MI), sepsis, septic shock, bleeding requiring transfusion, and/or progressive renal insufficiency.

### Statistical analysis

Preoperative patient characteristics including age, sex, race, BMI, and comorbidities and post-operative outcomes were compared across the three groups. Categorical variables were analyzed using Chi-Square or Fischer Exact tests. Continuous variables were analyzed using the Analysis of Variance (ANOVA) test. Multivariable logistic regression was performed to evaluate the impact of mFI-5 score on the odds of 30-day outcomes. Preoperative factors including age, sex, race, BMI, ASA score, smoking status, steroid use, bleeding disorders, and preoperative transfusions and gastrectomy type (total vs partial) were included in the regression model. All statistical analyses were performed using SPSS version 29 (IBM Corp., Amonk, NY, USA). *P*-values less than 0.05 were considered statistically significant.

## Results

### Patient demographics

A total of 7438 patients were included in this analysis. A comparison of preoperative characteristics between the three mFI-5 groups is summarized in Table 1. Higher mFI-5 score was associated with male gender (*p* = 0.004), older mean age (*p* < 0.001), black race (*p* < 0.001), higher median body mass index (BMI, *p* < 0.001), and higher American Society of Anesthesiologists (ASA) class (*p* < 0.001). Meanwhile, lower mFI-5 was associated with Hispanic ethnicity (*p* < 0.001) and tobacco use (*p* < 0.001). Preoperative steroid use, bleeding disorders, and transfusion requirements were more common in patients with higher mFI-5 scores. Furthermore, patients with higher frailty scores more commonly underwent partial gastrectomy compared to non-frail patients (*p* < 0.001).

**Table 1:** Comparison of patient characteristics between mFI-5 groups

		mFI-5 = 0 (*n* = 3032)	mFI-5 = 1 (*n* = 2805)	mFI-5 ≥ 2 (*n* = 1601)	*p*-value
Gender	Female	1335 (44.0%)	1136 (40.5%)	635 (39.7%)	**0.004**
	Male	1697 (56.0%)	1669 (59.5%)	965 (60.3%)	
Age (years)		59.6 (± 13.6)	69.1 (± 10.9)	70.6 (± 9.6)	** < 0.001**
Body Mass Index		24.8 (21.8–28.3)	26.3 (23.2–30.0)	28.2 (24.4–32.8)	** < 0.001**
Race	White	1544 (50.9%)	1440 (51.4%)	795 (49.7%)	** < 0.001**
	African American	340 (11.2%)	543 (19.4%)	334 (20.9%)	
	Asian	539 (17.8%)	383 (13.6%)	195 (12.2%)	
	Native American	37 (1.2%)	26 (0.9%)	13 (0.8%)	
	Hawaiian/Pacific Islander	16 (0.5%)	27 (0.9%)	11 (0.6%)	
	Other/Unknown	553 (18.3%)	381 (13.5%)	251 (15.6%)	
Hispanic (Y)		450 (14.8%)	295 (10.5%)	204 (12.7%)	** < 0.001**
ASA Classification	I	54 (1.8%)	4 (0.1%)	1 (0.1%)	** < 0.001**
	II	1075 (25.5%)	587 (20.9%)	148 (9.2%)	
	III	1771 (58.4%)	1973 (70.3%)	1218 (76.1%)	
	IV	124 (4.1%)	238 (8.5%)	230 (14.4%)	
	V	2 (0.1%)	0 (0.0%)	2 (0.1%)	
Tobacco Use		599 (19.8%)	484 (17.3%)	229 (14.3%)	** < 0.001**
Disseminated Cancer		274 (9.0%)	243 (8.7%)	151 (9.4%)	0.685
Steroid Use		122 (4.0%)	85 (3.0%)	83 (5.2%)	**0.002**
Bleeding Disorder		55 (1.8%)	67 (2.4%)	62 (3.9%)	** < 0.001**
Transfusion (72 h before surgery)		102 (3.4%)	125 (4.5%)	99 (6.2%)	** < 0.001**
Gastrectomy Type	Total	1164 (38.4%)	863 (30.8%)	419 (26.2%)	** < 0.001**
	Partial	1868 (61.6%)	1942 (69.2%)	1182 (73.8%)	

Continuous data expressed as mean ± standard deviation or median (interquartile range); categorical data represented as *n* (%)

Bolded values indicate significant differences defined as *p* < 0.005

*ASA *American Society of Anesthesiologists

### Post-operative outcomes

On unadjusted analysis (Table 2), increasing mFI-5 score was associated with higher rates of overall complications (*p* < 0.001) and serious complications (*p* < 0.001). There was no significant difference in rates of wound complications between groups. A step-wise increase in rates of pneumonia (4.9% to 8.1%, *p* < 0.001), unplanned intubation (1.7% to 5.5%, *p* < 0.001), failure to wean from the ventilator (2.0% to 4.7%, *p* < 0.001), ARF (0.3% to 1.4%, *p* < 0.001), urinary tract infection (2.0% to 3.1%, *p* = 0.048), CVA (0.1% to 0.6%, *p* = 0.017), cardiac arrect (0.5% to 1.5%, *p* = 0.001), MI (0.4% to 1.8%, *p* < 0.001), bleeding requiring transfusion (14.0% to 17.8%, *p* = 0.003), 30-day mortality (1.3% to 3.9%, *p* < 0.001), and non-home discharge (6.9% to 18.9%, *p* < 0.001) was observed with increasing mFI-5 score.

**Table 2:** Comparison of 30-day post-operative outcomes between mFI-5 groups

	mFI-5 = 0 (*n* = 3032)	mFI-5 = 1 (*n* = 2805)	mFI-5 ≥ 2 (*n* = 1601)	*p*-value
Overall complication	1714 (56.5%)	1706 (60.8%)	999 (62.4%)	** < 0.001**
Serious Complications	898 (29.6%)	925 (33.0%)	624 (39.0%)	** < 0.001**
Superficial SSI	111 (3.7%)	111 (4.0%)	78 (4.9%)	0.133
Deep SSI	26 (0.9%)	27 (1.0%)	17 (1.1%)	0.782
Organ Space SSI	191 (6.3%)	201 (7.2%)	111 (6.9%)	0.401
Wound Disruption	24 (0.8%)	28 (1.0%)	18 (1.1%)	0.496
Pneumonia	149 (4.9%)	169 (6.0%)	129 (8.1%)	** < 0.001**
Unplanned Intubation	51 (1.7%)	121 (4.3%)	88 (5.5%)	** < 0.001**
DVT	33 (1.1%)	56 (2.0%)	21 (1.3%)	**0.013**
PE	25 (0.8%)	21 (0.7%)	18 (1.1%)	0.414
Vent > 48 h	60 (2.0%)	107 (2.8%)	76 (4.7%)	** < 0.001**
Acute Renal Failure	9 (0.3%)	33 (1.2%)	22 (1.4%)	** < 0.001**
Urinary Tract Infection	60 (2.0%)	64 (2.3%)	50 (3.1%)	**0.048**
CVA	3 (0.1%)	10 (0.4%)	9 (0.6%)	**0.017**
Cardiac Arrest	14 (0.5%)	29 (1.0%)	24 (1.5%)	**0.001**
MI	11 (0.4%)	32 (1.1%)	29 (1.8%)	** < 0.001**
Transfusions	425 (14.0%)	418 (14.9%)	285 (17.8%)	**0.003**
Sepsis	145 (4.8%)	139 (5.0%)	85 (5.3%)	0.734
Septic Shock	60 (2.0%)	105 (3.7%)	48 (3.0%)	** < 0.001**
Return to OR	189 (6.2%)	204 (7.3%)	115 (7.2%)	0.238
Readmission	301 (9.9%)	276 (9.8%)	208 (13.0%)	**0.002**
30-day mortality	38 (1.3%)	86 (3.1%)	62 (3.9%)	** < 0.001**
Non-home discharge	206 (6.9%)	408 (14.7%)	300 (18.9%)	** < 0.001**

Continuous data expressed as mean ± standard deviation or median (interquartile range); categorical data represented as *n* (%)

Bolded values indicate significant differences defined as *p* < 0.005

*SSI* Surgical Site Infection, *DVT* Deep Vein Thrombosis, *PE* Pulmonary Embolism, Vent > 48 h = failure to wean from the ventilator > 48 h, *CVA* cerebrovascular accident/stroke with neurological deficit, *MI* myocardial infarction

### Multivariate analysis

Multivariable logistic regression was utilized to evaluate mFI-5 score as a possible predictor of various post-operative outcomes (Table 3). After adjusting for other factors, mild and moderate-to-severe frailty were found to be independent predictors of unplanned intubation (OR 2.06, CI 1.45–2.92, *p* < 0.001 vs OR 2.47 CI 1.68–3.62, *p* < 0.001), failure to wean from the ventilator (OR 1.68, CI 1.20–2.37, *p* = 0.003 vs OR 2.00, CI 1.36–2.95, *p* < 0.001), ARF (OR 3.25, CI 1.50–7.05, *p* = 0.003 vs OR 3.27, CI 1.42–7.55, *p* = 0.005), 30-day mortality (OR 1.73, CI 1.14–2.28, *p* = 0.009 vs OR 1.94, CI 1.23–3.05, *p* = 0.004), and non-home discharge (OR 1.34, CI 1.12–1.61, *p* = 0.001 vs OR 1.74, CI 1.42–2.13, *p* < 0.001). Relative to non-frail patients, moderate-to-severe frailty patients were more likely to experience overall complications (OR 1.43, CI 1.23–1.66, *p* < 0.001), serious complications (OR 1.42, CI 1.23–1.63, *p* < 0.001), pneumonia (OR 1.43, CI 1.09–1.89, *p* = 0.010), MI (OR 2.91, CI 1.38–6.14, *p* = 0.005), and readmission within 30 days (OR 1.33, CI 1.08–1.64, *p* = 0.008). Mild frailty was found to be a predictor of septic shock (OR 1.61, CI 11.14–2.28, *p* = 0.007).

**Table 3:** Multivariable regression analysis between mFI-5 group and post-operative outcomes

	mFI-5 = 1 vs 0			mFI-5 ≥ 2 vs 0		
	OR	95% CI	*p*-value	OR	95% CI	*p*-value
Overall Complications	1.09	0.96–1.24	0.193	1.43	1.23–1.66	** < 0.001**
Serious Complication	1.05	0.92–1.18	0.451	1.42	1.23–1.63	** < 0.001**
Pneumonia	1.06	0.83–1.35	0.657	1.43	1.09–1.89	**0.010**
Unplanned Intubation	2.06	1.45–2.92	** < 0.001**	2.47	1.68–3.62	** < 0.001**
DVT	1.43	0.90–2.28	0.131	0.80	0.44–1.46	0.459
Vent > 48 h	1.68	1.20–2.37	**0.003**	2.00	1.36–2.95	** < 0.001**
Acute Renal Failure	3.25	1.50–7.05	**0.003**	3.27	1.42–7.55	**0.005**
Urinary Tract Infection	0.92	0.62–1.36	0.659	1.26	0.82–1.94	0.300
CVA	2.14	0.56–0.82	0.269	2.68	0.66–10.87	0.168
Cardiac Arrest	1.49	0.74–3.00	0.269	1.73	0.81–3.66	0.155
MI	1.97	0.96–4.05	0.063	2.91	1.38–6.14	**0.005**
Transfusions	0.93	0.79–1.09	0.362	1.12	0.92–1.35	0.259
Septic Shock	1.61	1.14–2.28	**0.007**	1.29	0.84–1.96	0.246
Readmission	0.98	0.81–1.18	0.790	1.33	1.08–1.64	**0.008**
30-day mortality	1.73	1.14–2.62	**0.009**	1.94	1.23–3.05	**0.004**
Non-home discharge	1.34	1.12–1.61	**0.001**	1.74	1.42–2.13	** < 0.001**

Bolded values indicate significant differences defined as *p* < 0.005

*DVT*  Deep Vein Thrombosis; Vent > 48 h = failure to wean from the ventilator > 48 h; *CVA* cerebrovascular accident/stroke with neurological deficit, *MI* myocardial infarction

## Discussion

The mFI-5 score has previously been demonstrated to be a strong predictor of mortality and post-operative complications in many surgical sub-specialties [11–16]. In the present study, the mFI-5 score was evaluated as a predictor of 30-day post-operative complications following gastrectomy for gastric cancer. Patients with a high mFI-5 score were more likely to experience serious post-operative complications and early death compared to non-frail patients. Specifically, a high mFI-5 score was independently associated with higher rates of unplanned intubation, failure to wean from the vent within 48 h, acute renal failure, pneumonia, MI, and readmission.

These results are consistent with previous studies analyzing the association between frailty and outcomes following gastrectomy using other frailty measures. In a retrospective analysis performed by Zorbas et al., the mFI-11 was found to be an independent predictor of 30-day mortality, Clavien IV complications, and failure to rescue after a serious complication following non-bariatric gastrectomy [19]. Meng et al. demonstrated a significant, independent association between mFI-11 score and increased risk of pulmonary infections following radical gastrectomy for gastric cancer while Osaki et al. found mFI-11 to be a useful predictor of non-home discharge in gastric cancer patients undergoing gastrectomy [20, 21]. Jeong et al. demonstrated a relationship between frailty and post-gastrectomy mortality independent of other factors including age, sex, TNM stage, type of approach, gastrectomy type, and extent of lymph node dissection. In their study, the Study of Osteoporotic Fractures (SOF) index was used as a measure of frailty [22]. Utilizing the mFI-5, our study demonstrated similar associations between frailty and serious complications, cardiopulmonary complications, 30-day mortality, and non-home discharge, suggesting that the mFI-5 is comparably effective relative to other frailty metrics in predicting adverse post-operative outcomes in patients undergoing gastrectomy for gastric cancer.

However, compared to the mFI-11, the mFI-5 is less cumbersome due to its truncated nature, making it easier to calculate and use without sacrificing predictive ability. The mFI-5 also has several advantages over other factors utilized for preoperative risk assessment. While age and frailty are interrelated, age alone may not accurately reflect preoperative risk as patients with similar age may have markedly different physiologic reserve [9, 15]. The mFI-5 is multifactorial and may therefore represent a more complete picture of a patient’s physiologic condition prior to surgery. For example, our study found moderate-to-severe frailty to be a predictor of post-operative complications such as unplanned intubation, failure to wean from the ventilator for over 48 h, ARF, and MI, independent of other factors including age. Another metric often used to ascertain preoperative risk is ASA class. However, this is a subjective classification whereas the mFI-5 is an objective measure, mitigating the risk of interrater variability.

While there are not yet any prospective studies validating the use of the mFI-5 as a risk assessment tool in preoperative surgical patients, several studies have demonstrated the predictive capabilities of the mFI-5 for adverse outcomes following various surgeries and have highlighted the potential clinical usefulness of the score for risk stratification[11, 13, 16, 23–27]. Utilizing the mFI-5 as a preoperative risk assessment tool can help guide patient care by identifying patients with mild or moderate-to-severe frailty who may require additional presurgical discussions or optimization. Frailty results from the progressive, cumulative decline in functioning across multiple physiologic systems [9, 28]. These changes include decrease in cardiac and pulmonary function, weakened immune function, and altered drug metabolism secondary to multiple factors such as lower renal mass and function or reduced muscle mass [9, 10, 15]. Considering these physiologic alterations, efforts should be made proactively in preoperative planning to help mitigate the increased risks of adverse outcomes in patients with mild or moderate-to-severe frailty. These may include minimizing opioid use, deprescribing inappropriate or unnecessary medications, utilizing multidisciplinary care teams, preoperative nutritional optimization, and early involvement of physical therapy and exercise interventions post-operatively [15, 28, 29].

This study had several limitations due to several factors including the retrospective nature and data source. The data used is subject to input error. For example, patients were identified using CPT and ICD codes. Any errors in coding may result in failure to capture cases. Patients who were missing data for any of the mFI-5 variables were excluded, leading to possible selection bias. Furthermore, this study is limited to variables available in the NSQIP database. Important oncologic data such as TNM stage, degree of lymph node dissection, and histopathology, and factors such as mental acuity are not captured in the NSQIP data and could not be included when adjusting for confounders. Other relevant post-operative outcomes, such as anastomotic leak, are not recorded and therefore could not be analyzed in this study. Information regarding operative approach was not included in the NSQIP data analyzed and we were therefore unable to evaluate differences in outcomes based on open, laparoscopic, or robotic approach. Lastly, the NSQIP database only includes 30-day outcomes. Future studies should analyze the relationship between frailty and long-term outcomes.

## Conclusions

The mFI-5 is an independent predictor of overall and serious complications, 30-day mortality, and non-home discharge following gastrectomy for gastric cancer. The results of this study suggest that the mFI-5 may have utility as a concise, easy-to-use risk assessment tool for patients undergoing gastrectomy for gastric cancer, allowing surgeons to identify high-risk patients who may require enhanced pre-operative discussion and optimization and to mitigate the risk of adverse post-operative outcomes. However, further studies are needed to validate the mFI-5 as a risk stratification tool in a clinical setting.
